# Sutureless Biological Aortic Valve Replacement (Su-AVR) in Redo operations: a retrospective real-world experience report of clinical and echocardiographic outcomes

**DOI:** 10.1186/s12872-023-03652-7

**Published:** 2024-01-03

**Authors:** Ian Cummings, M Yousuf Salmasi, Halil Ibrahim Bulut, Alicja Zientara, Mahmoud AlShiekh, George Asimakopoulos

**Affiliations:** 1 Department of Cardiac Surgery, St Thomas Hospital, London, UK; 2 Department of Surgery, Imperial College London, QEQM Building, South Wharf Road, London, UK; 3 Department of Cardiothoracic Surgery, Royal Brompton and Harefield NHS Foundation Trust, London, UK

**Keywords:** Perceval, Sutureless, Aortic valve replacement, Redo cardiac surgery

## Abstract

**Objective:**

This retrospective study aimed to compare the outcomes of sutureless aortic valve replacement (su-AVR) and conventional bioprosthetic sutured AVR (cAVR) in high-risk patients undergoing redo surgery.

**Methods:**

A total of 79 patients who underwent redo AVR between 2014 and 2021 were included in the study. Of these, 27 patients underwent su-AVR and 52 underwent cAVR. Patient characteristics and clinical outcomes were analysed using multivariate regression and Kaplan Meier survival test.

**Results:**

The groups were similar in terms of age, gender, left ventricular function, and number of previous sternotomies. In cases of isolated AVR, su-AVR had significantly lower cross clamp times than cAVR (71 vs. 86 min, *p* = 0.03). Postoperatively, 4 cAVR patients required pacemaker compared to zero patients in the su-AVR group. There were no significant differences between the two groups in terms of postoperative complications, intrahospital stay (median 9 days, IQR 7–20), or in-hospital mortality (1 su-AVR; 2 cAVR). The long-term survival rate was similar between the su-AVR (90%) and cAVR (92%) groups (log rank *p* = 0.8). The transvalvular gradients at follow-up were not affected by the type of valve used, regardless of the valve size (coef 2.68, 95%CI -3.14–8.50, *p* = 0.36).

**Conclusion:**

The study suggests that su-AVR is a feasible and safe alternative to cAVR in high-risk patients undergoing redo surgery. The use of su-AVR offers comparable outcomes to cAVR, with reduced cross clamp times and a lower incidence of postoperative pacemaker requirement in isolated AVR cases. The results of this study contribute to the growing body of evidence supporting the use of su-AVR in high-risk patients, highlighting its feasibility and safety in redo surgeries.

## Introduction

As the age of recipients of bioprosthetic aortic valve replacements (AVR) continues to decrease, we are witnessing a rise in mid-term and late AVR prosthetic dysfunction cases. The widespread practice of redo-surgical access and replacement of the failed AVR with another sutured valve prosthesis or root replacement is complex and requires careful patient and prosthesis selection [1, 2]. Transcatheter technology is also emerging as an alternative to treat cases of bioprosthetic AVR failure, eliminating the need for sternal re-entry and its associated complications [3, 4]. However, in younger, fitter patients with early valve failure caused by accelerated leaflet calcification or prosthetic valve endocarditis, surgical explanation of the prosthetic valve and repeat implantation of a new prosthesis remains highly advantageous.

The selection of a suitable prosthesis during complex redo AVR procedures depends on various factors, including the patient's age, comorbidities, and lifestyle, as well as technical aspects such as aortic access, annular size, shape, and whether it can accept a Perceval. The index procedure is also vital to consider implanting a Perceval within a previous root replacement (including homograft) would be different from implanting within a native root, and different still from a previous stented valve.

In this regard, providing patients with technological "upgrades" in the second, third, or even fourth AVR may be necessary, particularly if there are potential technical advantages. The advantages of using SU-AVR include the avoidance of extensive tissue damage during explanation of the old valve ring, as well as the prospect of fast and simple implantation, which reduces the risk of cardiac ischemia and cardiopulmonary bypass, particularly in a long and protracted redo case. This has the potential to decrease post-operative complications and improve patient recovery.

As noted, while the use of SU-AVR in redo-AVR procedures may have potential advantages over conventional sutured bioprostheses, there are also potential disadvantages to consider [5]. One of these is the need for a higher aortotomy, which may not be achievable in a redo-sternotomy[5]. Additionally, the long-term outcomes of Perceval valves in redo-AVR procedures are not yet clear, particularly with regard to the incidence of paravalvular leak. However, despite these uncertainties, some published case reports have shown promising outcomes when using sutureless or SU-AVRs in complex redo procedures, particularly for replacing degenerated stentless bioprostheses and homografts[5]. Nonetheless, further studies are needed to understand the potential benefits and drawbacks of these approaches more fully, particularly compared to conventional sutured bioprostheses[5].

To this end, the current study aims to contribute to the existing body of knowledge on SU-AVR by reporting our experience using the Perceval valve for redo-AVR procedures. Our analysis will consider short and mid-term outcomes and compare the performance of the Perceval valve with that of conventional sutured bioprostheses. Ultimately, our findings may help to inform future clinical decision-making regarding the optimal selection of prostheses for patients undergoing redo-AVR procedures.

## Methods

### Study design and ethical evaluation

Perioperative data was retrospectively analysed from a prospectively collated database at a single cardiothoracic institution between 2014 and 2021. Due to the retrospective nature of the study, the requirement for ethical approval was waivered by the Research Ethics Office at the Royal Brompton and Harefield Foundation Trust.

### Inclusion criteria

Patients were included if they fulfilled two important criteria: i) they had previously undergone aortic valve surgery (non-repair) in adulthood more than 6 months prior to their inclusion in the study; and ii) they were undergoing re-sternotomy and reimplantation of a new aortic valve prosthesis.

Patients who had received both mechanical and biological valves in the prior index procedure were included. Prior aortic root replacements were also included, including homograft procedures.

All indications for redo aortic valve intervention were considered, including structural valve degeneration and endocarditis. Patients were divided into two groups according to the type of AVR prosthesis they were receiving: either i) a sutureless (SU-AVR) Perceval valve; or ii) a Perimount Magnaease prosthesis as a conventional sutured valve (cAVR).

Patients receiving other types of rapid deployment valves were excluded. To ensure a consistent comparison between the groups, other marketed conventional sutured valves, although used in our institution, were excluded from the study.

### Patient selection for REDO SAVR

In the strategic planning of reoperations within our hospital, several crucial factors were meticulously considered to tailor the approach for each patient. These factors encompassed the status of the index valve, including calcification and infection status, as well as the potential risk of coronary sinus sequestration—a complication associated with valve-in-valve procedures. Additionally, the patients' life expectancy was a pivotal determinant in the decision-making process.

In alignment with these parameters, patients were judiciously directed towards either valve-in-valve transcatheter aortic valve replacement (TAVR), mechanical prosthesis, or bioprosthesis interventions. Notably, a noteworthy proportion of individuals within this series were deemed unsuitable for valve-in-valve procedures due to specific characteristics of their index valves. Particularly, those with biological index valves underwent surgical aortic valve replacement for diverse reasons, reflecting the nuanced and individualized nature of the decision-making process in this cohort.

### Procedure

All reoperations were performed via median sternotomy, with the use of an oscillating saw. The strategy of cannulation for cardiopulmonary bypass was patient-specific and subject to the surgeon’s discretion. In general, if vital mediastinal structures were not at risk from sternal re-entry (as assessed from pre-operative cross-sectional imaging, sternal re-entry followed by standard central cannulation was the first-line desirable method. However, femoral cannulation was performed prior to sternotomy i) injury to vital structures was of concern when planning sternotomy on pre-operative cross-sectional imaging; and ii) femoral vessels were of suitable calibre and amenable to cannulation. Once CPB was initiated, systemic normothermia or mild hypothermia (32 °C) if a patent mammary artery bypass was present. Once the aorta was cross clamped, and the heart successfully arrested, myocardial protection was achieved with intermittent antegrade blood cardioplegia (including direct ostial infusion), with or without retrograde cardioplegia, if the coronary sinus was accessible during initial myocardial dissection.

The decision regarding prosthesis or root replacement was made at the surgeon's discretion. In cases where a Perceval valve was chosen, aortotomy was typically performed through a high transverse incision. For patients determined for Perceval implantation, meticulous and precise decalcification of the aortic root was undertaken. The valve was positioned optimally using three guide sutures and then implanted. Post-implantation, no balloon expanding system was utilized; only the valve's functionality was assessed. In instances of neo-sinus/fistulating root endocarditis, patch repair of the aortic root was implemented. Concurrent procedures followed standard techniques.

### Echocardiographic assessment

Echocardiographic assessments were conducted by seasoned non-invasive cardiologists within the echocardiographic department of our institution. In tandem with a comprehensive preoperative evaluation encompassing the left ventricle and aortic valve, subsequent postoperative examinations included valve imaging and assessment of left ventricular function. These evaluations were performed within 30 days post-surgery and subsequently repeated at a median interval of 24 months during the post-discharge period. Importantly, continuity and standardization were ensured by entrusting these evaluations consistently to the same proficient team within our institutional framework.

### Data collection

The cardiac surgical database is locally managed and centrally overseen at a national level, following national guidelines for minimal perioperative data collection, including pre-operative co-variates, detailed operative characteristics and post-operative care, including the record of short-term complications. Early and mid-term outcomes were assessed based on echocardiographic findings and grading of aortic regurgitation.

### Statistical analysis

The results underwent analysis and were presented in terms of means and standard deviations. Normal distribution of pre-operative covariates was assessed using the Shapiro–Wilk test. Between-group characteristics were evaluated for statistical differences using either the Student T test or the Wilcoxon Rank Test for non-parametric variables. Multivariate regression models were developed to examine the influence of various covariates on both short- and long-term outcomes. The selection of multivariate regression test parameters was selected by the clinical expertise of the academic surgical team. Additionally, Kaplan–Meier survival analyses with Log-rank tests were employed to determine overall survival, and adjusted odds ratios with 95% confidence intervals (CI) for binary outcomes were calculated. The statistical analyses were conducted using Jamovi (Version 2.4) [Computer Software].

## Results

A total of 79 patients who underwent redo AVR between 2014 and 2021 were included in the study. Of these, 27 patients underwent su-AVR and 52 underwent cAVR.

### Patient characteristics

Table 1 presents a comprehensive comparison between the su-AVR group and the cAVR group. In terms of demographics, there was no significant difference observed in the mean age between the cAVR (57.6 ± 2.5 years) and su-AVR (59.6 ± 2.4 years) groups (*P* = 0.607). Similarly, gender distribution displayed no significant difference, with 36 males in the cAVR group and 16 males in the su-AVR group (*P* = 0.379). The Body Mass Index (BMI) for patients in the cAVR group was 26.6 ± 0.7, while the su-AVR group had a slightly higher BMI of 28.7 ± 1.0 (*P* = 0.081).

**Table 1:** Pre-operative patient characteristics comparing cAVR and su-AVR groups

	Conventional AVR (cAVR) (Perimount) (*n* = 52)	Sutureless AVR (su-AVR) (Perceval) (*n* = 27)	*P* value
***Demographics***			
Age	57.6 ± 2.5	59.6 ± 2.4	0.607*
Male	69.2 (36)	59.2 (16)	0.379
BMI	26.6 ± 0.7	28.7 ± 1.0	0.081*
**Cardiac-Comorbidities**			
Hypertension	55.8 (29)	66.7 (18)	0.352
PAD	3.8(2)	7.4 (2)	0.496
Infective Endocarditis	17.3 (9)	29.6 (8)	0.252
**NYHA(median)**	**3**	**2**	**0.036**
More than 1 sternotomy	7.7 (4)	22.2 (6)	0.250
***Other-Comorbidities***			
Diabetes Oral	5.8 (3)	14.8 (4)	0.542
Diabetes Insulin	3.8 (2)	0.0 (0)	0.542
Smoking	42.3 (22)	48.1 (13)	0.555
COPD	9.6 (5)	22.2 (6)	0.127
History of stroke	5.8 (3)	7.4 (2)	0.816
CKD	3.8 (2)	7.4 (2)	0.514
***Echocardiography Findings***			
***Left Ventricular Functions***			0.394
EF good	88.5 (46)	81.4 (22)	
EF moderate	9.6 (5)	14.8 (4)	
EF poor	1.9 (1)	3.8 (1)	
EF%	57.5 ± 1.3	58.2 ± 2.0	0.742
***Valvular Functions***			0.527
Aortic Stenosis	23.1 (12)	33.3 (9)	
Aortic Regurgitation	59.6 (31)	44.4 (12)	
Aortic Mixed Pathology	11.5 (6)	18.3 (5)	
***Reason for Reoperation***			*0.067*
Prosthetic Failure	36.5 (19)	48.1 (13)	
Infection	15.4 (8)	29.6 (8)	
Thrombosis	7.7 (4)	14.8 (4)	
Paravalvular leak	0.0 (0)	3.8 (1)	
Native valve degradation	3.8 (2)	3.8 (1)	
Regurgitation	19.2 (10)	0.0 (0)	

Regarding cardiac comorbidities, the presence of hypertension was observed in 29 patients from the cAVR group and 18 patients from the su-AVR group, with no significant difference in distribution (*P* = 0.352). Peripheral Arterial Disease (PAD) was reported in 2 patients in both the cAVR and su-AVR groups, with no significant disparity (*P* = 0.496). A comparable number of cases of Infective Endocarditis were seen in the cAVR (9 patients) and su-AVR (8 patients) groups (*P* = 0.252). The New York Heart Association (NYHA) classification demonstrated a median of 3 (range 2–3) in the cAVR group and a median of 2 (range 1–3) in the su-AVR group, showing a statistically significant difference (*P* = 0.036). The occurrence of more than one previous sternotomy was noted in 4 patients in the cAVR group and 6 patients in the su-AVR group, with no significant distinction (*P* = 0.250).

In terms of the other comorbidities, the prevalence of Oral Diabetes was recorded in 3 patients from the cAVR group and 4 patients from the su-AVR group (*P* = 0.542). No cases of Insulin-dependent Diabetes were observed in the su-AVR group, while 2 cases were reported in the cAVR group (*P* = 0.542). Smoking was found in 22 patients from the cAVR group and 13 patients from the su-AVR group, with no significant discrepancy (*P* = 0.555). Chronic Obstructive Pulmonary Disease (COPD) was documented in 5 patients in the cAVR group and 6 patients in the su-AVR group, showing no significant difference (*P* = 0.127). A history of stroke was reported in 3 patients from the cAVR group and 2 patients from the su-AVR group, with no significant variance (*P* = 0.816). Chronic Kidney Disease (CKD) was observed in 2 patients from both the cAVR and su-AVR groups, displaying no significant difference (*P* = 0.514).

Regarding the preoperative echocardiographic findings, the assessment of left ventricular functions demonstrated no significant difference between the groups. In the Conventional Aortic Valve Replacement (cAVR) group, 46 patients exhibited good left ventricular ejection fraction (EF), while 5 patients had moderate EF, and 1 patient had poor EF. Similarly, Sutureless Aortic Valve Replacement (su-AVR) group showed 22 patients with good EF, 4 patients with moderate EF, and 1 patient with poor EF. The mean EF percentage was 57.5 ± 1.3 in the cAVR group and 58.2 ± 2.0 in the su-AVR group, with no statistically significant difference observed between the two groups (P = 0.742). In the cAVR group, 12 patients exhibited aortic stenosis, 31 patients had aortic regurgitation, and 6 patients had aortic mixed pathology. In the su-AVR group, 9 patients showed aortic stenosis, 12 patients had aortic regurgitation, and 5 patients had aortic mixed pathology. No significant differences in valvular functions were noted between the two groups (*P* = 0.527).

In terms of the reasons for reoperation, there were no substantial discrepancies between the cAVR and su-AVR groups. In the cAVR group, 19 patients required reoperation due to prosthetic failure, 8 patients due to infection, 4 patients due to paravalvular leak, 1 patient due to thrombosis, 2 patients due to native valve degradation, and 10 patients due to regurgitation. In the su-AVR group, 13 patients underwent reoperation for prosthetic failure, 8 patients for infection, 4 patients for paravalvular leak, 1 patient for thrombosis, 1 patient for native valve degradation, and none for regurgitation. The differences in reasons for reoperation did not reach statistical significance between the two groups (*P* = 0.067).

### Operative parameters

The Table 2 showcases the varying types of explants within each group. In the cAVR group, there were 6 mechanical valve explants, 22 biological valve explants, 19 native valve explants, 2 autograft explants, 1 homograft explant, and 2 freestyle explants. The su-AVR group displayed 5 mechanical valve explants, 17 biological valve explants, 1 native valve explant, 4 homograft explants, and no cases of autograft or freestyle explants.

**Table 2:** Valve explant types

	Conventional AVR (cAVR) (Perimount) (*n* = 52)	Sutureless AVR (su-AVR) (Perceval) (*n* = 27)
Mechanical	11.5 (6)	18.3 (5)
Biological	42.3 (22)	63.0 (17)
Native	36.5 (19)	3.8 (1)
Autograft	3.8 (2)	0.0 (0)
Homograft	1.9 (1)	14.8 (4)
Freestyle	3.8 (2)	0.0 (0)

The Table 3 demonstrates intraoperative outcomes. The cAVR group exhibited a CPB time of 168.4 ± 11.1 min, while the su-AVR group had a CPB time of 165.9 ± 19.6 min (*P* = 0.452). Regarding XC time, the cAVR group had a mean time of 108.8 ± 6.3 min, while the su-AVR group demonstrated a mean time of 92.0 ± 8.6 min (*P* = 0.124). In the cAVR group, 36 cases were elective, 15 were urgent, and 1 was emergency, while in the su-AVR group, 17 cases were elective, 10 were urgent, and none were emergency, displaying no significant difference (*P* = 0.622). The cAVR group included 12 cases with a valve size of 21, 21 cases with size 23, 14 cases with size 25, and 5 cases with size 27. In contrast, the su-AVR group comprised 10 cases with size 21, 13 cases with size 23, 3 cases with size 25, and 1 case with size 27. A statistically significant difference was noted in valve size distribution between the groups (*P* = 0.048).

**Table 3:** Operative parameters

	Conventional AVR (cAVR) (Perimount) (*n* = 52)	Sutureless AVR (su-AVR) (Perceval) (*n* = 27)	*P*-value
CPB time	168.4 ± 11.1	165.9 ± 19.6	0.452
AXC time	108.8 ± 6.3	92.0 ± 8.6	0.124
**Operative urgency**			0.622
Elective	69.2 (36)	63.0 (17)	
Urgent	28.8 (15)	37.0 (10)	
Emergency	2.0 (1)	0.0 (0)	
**Valve size**			**0.048**
21	23.1 (12)	37.0 (10)	
23	40.4 (21)	48.2 (13	
25	26.9 (14)	11.1 (3)	
27	9.6 (5)	3.8 (1)	

### Postoperative complication outcomes

In-hospital mortality was found to be similar between the two groups. Specifically, there were 2 deaths out of 52 patients (3.8%) in the cAVR group and 1 out of 27 patients (3.7%) in the su-AVR group. This difference was not statistically significant (*P* > 0.05). The incidence of stroke was lower in the cAVR group, but this difference was not statistically significant (1 out of 52 patients (1.9%) in the cAVR group vs. 3 out of 27 patients (11.1%) in the su-AVR group, *P* > 0.05). No cases of gastrointestinal bleeding were observed in either group, which was attributed to the use of biologic prostheses and the absence of anti-coagulation. However, the su-AVR group had lower rates of respiratory complications (7.4% vs. 19.2%, *P* > 0.05), the need for dialysis (0% vs. 7.7%, *P* > 0.05), and pericardial/pleural effusions (3.7% vs. 15.3%, *P* > 0.05) compared to the cAVR group. In addition, none of the patients in the su-AVR group required postoperative pacemaker implantation, while this was necessary in 4 out of 52 patients (7.7%) in the cAVR group. However, this difference was not statistically significant (*P* > 0.05) (Table 3).

### Echocardiographic outcomes

**Fig. 1:**
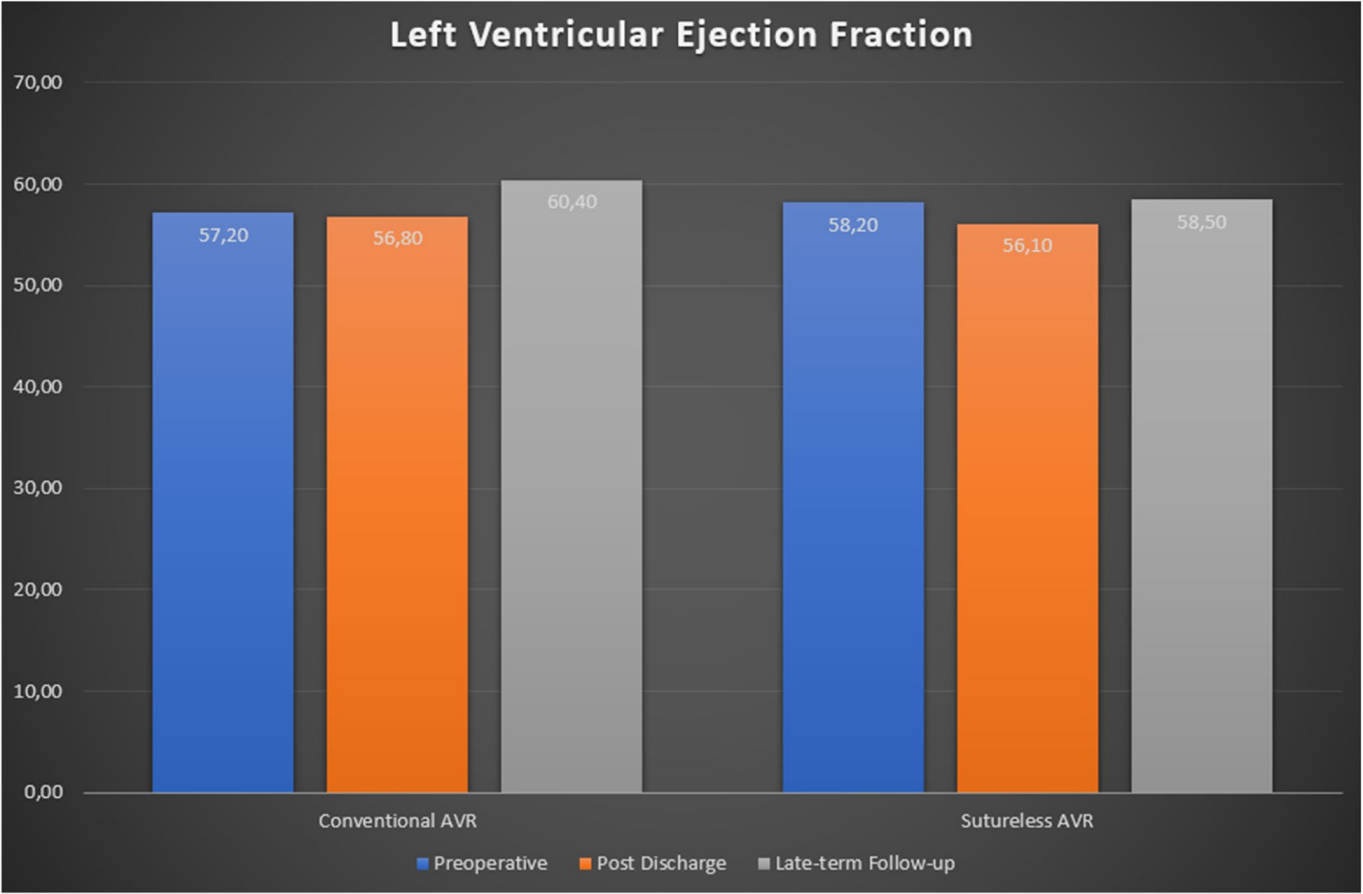
Left ventricular ejection fraction improvement at median 24-month follow-up

**Fig. 2:**
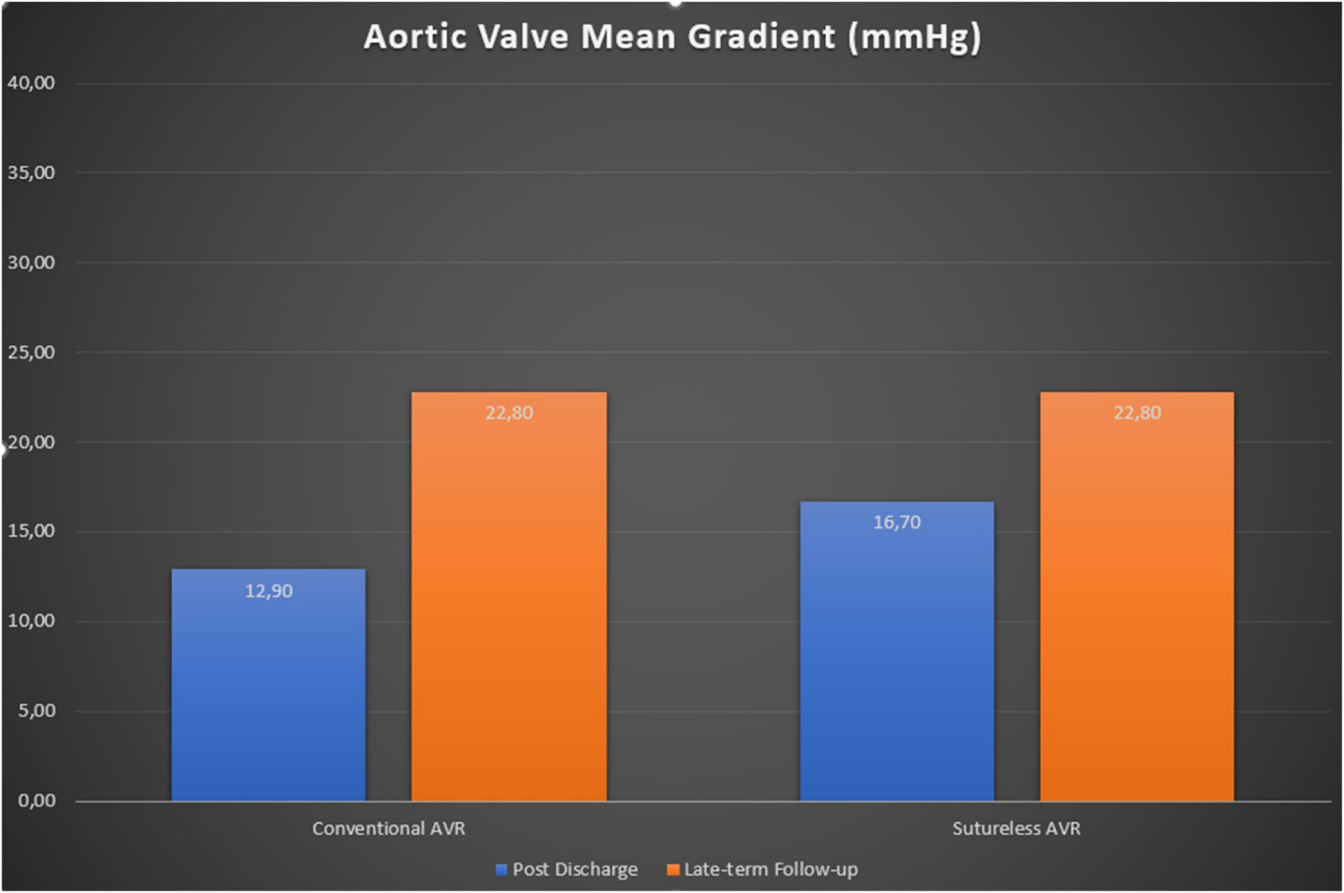
Aortic Valve Gradient after Redo AVR

Table 5 provides a comprehensive analysis of postoperative echocardiographic outcomes between the Conventional Aortic Valve Replacement (cAVR) and Sutureless Aortic Valve Replacement (su-AVR) groups (Table 5, Figs. 1, 2).

### Post discharge

Left Ventricular Functions displayed no significant differences between groups. In the cAVR group, 31 patients demonstrated good ejection fraction (EF), 9 patients had moderate EF, and 1 patient had poor EF. Similarly, in the su-AVR group, 19 patients exhibited good EF, 4 had moderate EF, and 1 had poor EF. The mean EF percentages were 56.8 ± 10.0 in the cAVR group and 56.1 ± 11.4 in the su-AVR group (*P* = 0.537). Prosthesis Functions indicated comparable results. The cAVR group had 34 normally functioning prostheses, 6 with mild aortic regurgitation (AR), 1 with moderate AR, and none with severe AR. Likewise, the su-AVR group had 21 normally functioning prostheses, 2 with mild AR, 1 with moderate AR, and none with severe AR (*P* = 0.714). Aortic Valve Gradient showed variations. Peak gradients were 23.2 ± 10.6 mm Hg in the cAVR group and 30.5 ± 14.8 mm Hg in the su-AVR group (*P* = 0.026). Mean gradients were 12.9 ± 6.4 mm Hg in the cAVR group and 16.7 ± 8.4 mm Hg in the su-AVR group (*P* = 0.045). The distribution of gradients under 20 mm Hg, 20–39 mm Hg, and 40 + mm Hg varied between the groups.

### Late term follow-up (median 24 months)

Late Term Follow-up revealed comparable findings. Left Ventricular Functions showed no significant differences, with 31 patients with good EF in the cAVR group and 18 in the su-AVR group. EF percentages were 60.4 ± 6.8 in the cAVR group and 58.5 ± 9.2 in the su-AVR group (*P* = 0.391). The cAVR group had 29 normally functioning prostheses, 4 with mild AR, none with moderate AR, and none with severe AR. The su-AVR group had 14 normally functioning prostheses, 4 with mild AR, 2 with moderate AR, and none with severe AR (*P* = 0.116). Aortic Valve Gradient values were similar. Peak gradients were 12.9 ± 7.1 mm Hg in the cAVR group and 15.6 ± 13.6 mm Hg in the su-AVR group (*P* = 0.360). Mean gradients were 22.8 ± 12.3 mm Hg in the cAVR group and 22.8 ± 13.6 mm Hg in the su-AVR group (*P* = 0.997). The distribution of gradients within specified ranges also showed similarities between the groups.

### Survival

**Fig. 3:**
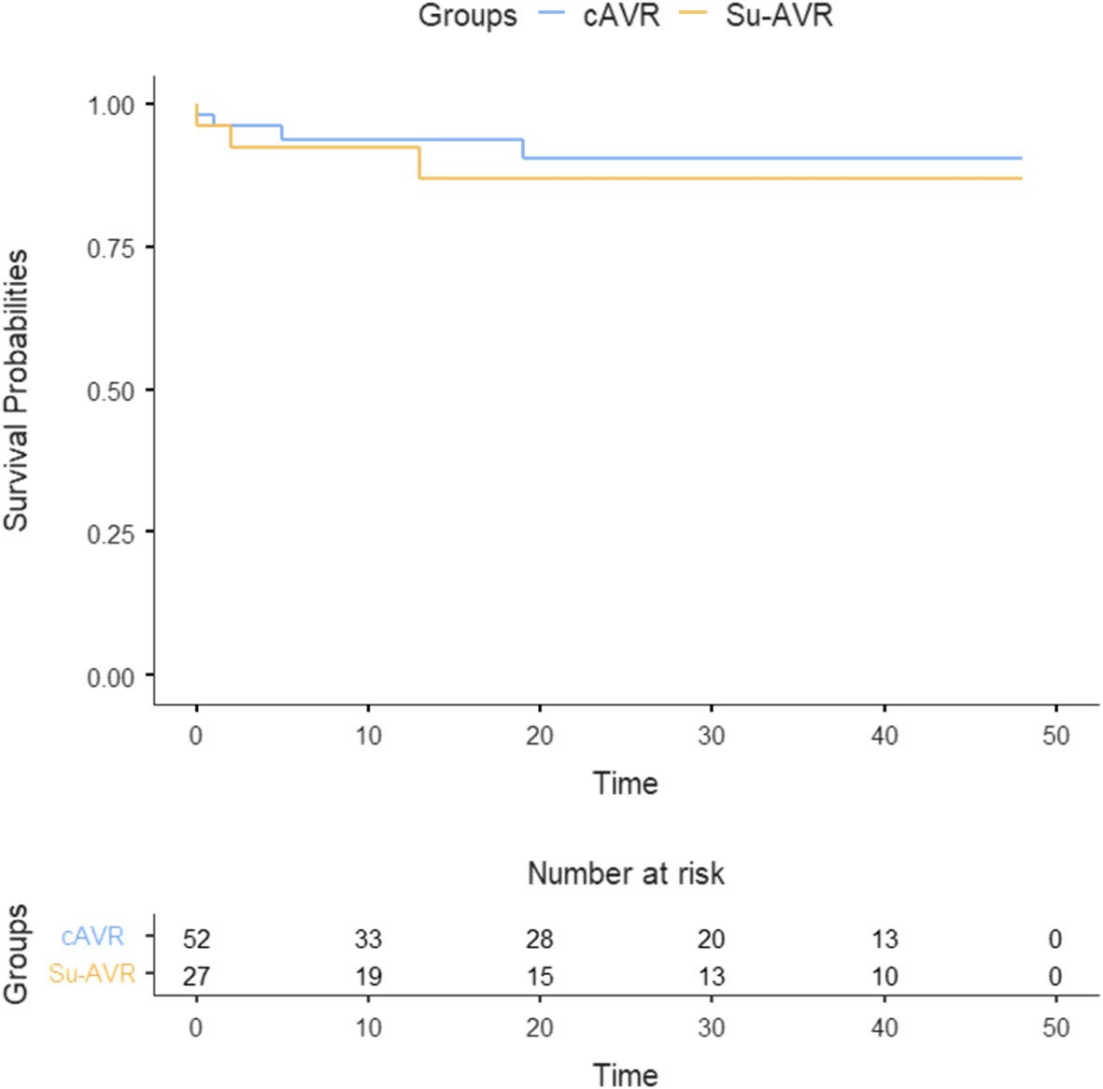
Kaplan–Meier survival curve

Kaplan Meier analysis revealed similar survival rates between the su-AVR and cAVR groups, (crude log rank test *p* = 0.315) (Fig. 3). A multivariate regression analysis was conducted to assess for predictors of early-term survival, including the choice of valve, age, gender, and pre-operative LV function. The results identified no significant predictors of long-term survival (*p* > 0.05). Specifically, the choice of valve did not influence survival (hazard ratio 1.22, 95% CI 0.14 – 10.25, *p* = 0.855). Furthermore, multivariate regression test was employed to determine, predictors of late-term survival. Age, NYHA, Perceval, smoking status, COPD, gender, and HT did not show statistical significance for late-term survival. However, patients with Type 2 Diabetes Mellitus (T2DM) demonstrated a significantly higher hazard ratio of 15.1 (95% CI: 1.593–142.424, *p* = 0.018), highlighting it as a substantial risk factor for reduced survival.These findings are summarized in Tables 4, 5, 6 and 7.

**Table 4:** Early term post-operative outcomes

	Conventional AVR (cAVR) (Perimount) (*n* = 52)	Sutureless AVR (su-AVR) (Perceval) (*n* = 27)	*P*-value
Pulmonary complications	19.2 (10)	7.4 (2)	0.164
Stroke	1.9 (1)	11.1 (3)	0.073
Transient Ischemic Attack	0.0 (0)	0.0 (0)	-
Dialysis needing	7.7 (4)	0.0 (0)	0.293
Gastrointestinal bleeding	0.0 (0)	0.0 (0)	-
Effusion	15.4 (8)	3.8 (1)	0.121
In hospital mortality	3.8 (2)	3.8 (1)	0.974

**Table 5:** Postoperative Echocardiographic outcomes

	Conventional AVR (cAVR) (Perimount) (*n* = 52)	Sutureless AVR (su-AVR) (Perceval) (*n* = 27)	*P*-value
***Post Discharge***			
***Left Ventricular Functions***			0.826
EF good	75.6 (31)	79.1 (19)	
EF moderate	22.0 (9)	16.7 (4)	
EF poor	2.4 (1)	4.2 (1)	
EF%	56.8 ± 10.0	56.1 ± 11.4	0.537
***Prosthesis Functions***			0.714
Normally functioning	82.8 (34)	87.5 (21)	
Mild AR	14.6 (6)	8.3 (2)	
Moderate AR	0.0 (0)	4.2 (1)	
Severe AR	0.0 (0)	0.0 (0)	
**Aortic Valve Gradient**			
**Peak**	23.2±10.6	30.5±14.8	**0.026**
**Mean**	12.9±6.4	16.7±8.4	**0.045**
Under 20 mm Hg	85.4 (35)	75.0 (18)	
20–39 mm Hg	14.6 (6)	25.0 (6)	
40- mm Hg	0.0 (0)	0.0 (0)	
**Late Term Follow-up (median 24 month)**			
***Left Ventricular Functions***			
EF good	93.9 (31)	90.0 (18)	
EF moderate	6.1 (2)	10.0 (2)	
EF poor	0.0 (0)	0.0 (0)	
EF%	60.4±6.8	58.5±9.2	0.391
***Prothesis Functions***			0.116
Normally functioning	87.9 (29)	70.0 (14)	
Mild AR	12.1 (4)	20.0 (4)	
Moderate AR	0.0 (0)	10.0 (2)	
Severe AR	0.0 (0)	0.0 (0)	
**Aortic Valve Gradient**			
Peak	12.9±7.1	15.6±13.6	0.360
Mean	22.8±12.3	22.8±13.6	0.997
Under 20 mm Hg	48.4 (15)	52.6 (10)	
20–39 mm Hg	41.9 (13)	42.1 (8)	
40- mm Hg	9.7 (3)	5.3 (1)

**Table 6:** Multivariate regression analysis: impact of patient covariates and valve prosthesis on patient early term survival (30-day)

	Hazard ratio	Standard error	95% CI	*P* value
Perceval	1.22	1.32	0.14 – 10.25	0.855
Age	1.03	0.04	0.95 – 1.10	0.475
Gender	0.72	0.74	0.10 – 5.40	0.752
BMI	0.95	0.10	0.76 – 1.17	0.610
Smoking	1.43	1.17	0.29 – 7.14	0.663
LVEF	0.95	0.04	0.88 – 1.02	0.147

**Table 7:** Multivariate regression analysis: impact of patient covariates and valve prosthesis on patient Late-term survival (median 24-month)

	Hazard ratio	Standard error	95% CI	*P* value
Age	1.1	0.047	0.976–1.172	0.149
**T2DM**	**15.1**	**1.1**	**1.593–142.424**	**0.018**
Smoking	0.1	1.5	0.005–1.361	0.080
COPD	1.1	1.5	0.057–21.364	0.948
Gender	7.5	1.6	0.325–175.942	0.207
HT	0.1	1.3	0.008–1.304	0.079
Perceval	3.2	1.3	0.264–41.189	0.354
NYHA	1.0	0.7	0.264–4.090	0.957

## Discussion

This study is the first to directly compare the outcomes of redo-AVR procedures between the Perceval valve and an established sutured bioprosthesis. The results of the study indicate that there were very similar early- and late-term outcomes between the two valve choices, suggesting that the Perceval valve may be a viable option for high-risk patients who require redo-AVR.

The utilization of su-AVR continues to provide the well-established advantage it offers in initial cardiac surgeries, resulting in reduced cardiopulmonary bypass and aortic cross-clamp durations. This has been consistently linked with decreased early-term mortality and a shorter duration of hospitalization in extensive databases [6] [7], making it an attractive option for high-risk operations such as redo AVRs.

### Pacemaker implantation after the procedure

Our study highlights a crucial finding regarding the lower rate of pacemaker implantation in the su-AVR group. Prior reports have indicated that su-AVR, particularly with the Perceval valve, is associated with a higher incidence of postoperative pacemaker implantation in first-time AVR surgery [8, 9]. Although this has been attributed to the initial part of the learning curve, where over-sizing may be more common, this complication is significant and cannot be overlooked [8, 9]. However, our research suggests that precise suture placement during cAVR is more challenging in cases where the annulus is already distressed and fibrosed, and the use of su-AVR may help circumvent this issue.

Interestingly, our study found a higher proportion of small valves were used in the su-AVR group compared to cAVR; this may explain the disparity in PPM results [10]. It's important to note that in our practice, we do not utilize balloon expansion after sutureless valve implantation, a factor we believe might contribute to our success in mitigating PPM incidence. Overall, our findings suggest that su-AVR may provide benefits in reducing the incidence of pacemaker implantation, especially in patients with a fibrosed annulus.

### A higher risk patient cohort

Redo AVR has traditionally been associated with a higher rate of complications compared to first-time SAVR in the literature, with reported operative mortality rates ranging between 4 and 9% [1] [11]. The formation of adhesions and loss of tissue planes between the heart, mediastinal structures, and sternum after the index SAVR increases the risk of complications during sternal re-entry and dissection, particularly in patients who are older or have comorbidities [2]. These risks are further compounded by the proximity to critical thoracic structures. The sequelae of redo surgical AVR may include stroke, myocardial infarction, new atrial fibrillation, and permanent pacemaker implantation.

In a meta-analysis by Formica et al., valve-in-valve transcatheter replacement was found to be advantageous in terms of complications compared to redo aortic valve surgery but redo aortic valve replacement surgery had advantages in medium and long-term survival [12]. In our study, there was no significant difference in perioperative complications between the su-AVR and cAVR groups. However, there was a reduced frequency of effusion and in-hospital mortality in the su-AVR group. Furthermore, none of the patients in the su-AVR group required dialysis, whereas dialysis was needed in four patients in the cAVR group. This suggests that the reduced operative time afforded by su-AVR may have benefits for reducing post-operative coagulopathy, metabolic disturbance, and ICU complications in redo AVR procedures. Much of this may be attributed to the shorter cross-clamp and CPB times.

### Long-term benefit

In the absence of long-term data on valve durability, the use of su-AVR in patients undergoing redo cardiac surgery is not typically indicated, due to the complexity of the procedure and the associated risk of perioperative complications and mortality.

The choice of bioprosthetic AVR variant has not been found to offer a significant survival advantage, but su-AVR has demonstrated some advantages in high-risk patient groups. Hanedan et al. found that su-AVR was three times more advantageous than cAVR in terms of early-term survival in high-risk patients, although the difference was statistically insignificant (94.7% vs 84.6%) [13]. Similarly, Salmasi et al.'s meta-analysis on su-AVR vs cAVR did not find a significant difference in bleeding and early-term survival, but su-AVR's shorter operative time was advantageous with a shorter ICU stay [14]. Coti et al.'s su-AVR study of 700 cases reported a three-year and five-year survival rate of 91% and 76%, respectively, demonstrating that su-AVR was feasible for mid-term survival, even in redo cardiac surgery patients [15]. Our study supports these findings, showing that su-AVR is comparable to cAVR in terms of short/mid/long-term survival in redo surgeries. While the choice of bioprosthetic AVR variant may not significantly affect survival rates, su-AVR's shorter operative time may lead to fewer complications and improved outcomes.

### Endocarditis

Prosthetic valve endocarditis is a highly morbid condition and compounds the risk of redo surgery according to most risk-scoring systems. The incidence can be as high as 16% in patients with prior AVR [16] and patients who require redo-surgery have exceptionally high morbidity and mortality rates [17] [18]. Complex procedures, such as prolonged cardiopulmonary bypass time and cross-clamp time, are often required, and these are associated with increased mortality and severe perioperative complications [19].

In a recent study, 29.6% of the su-AVR group and 17.3% of the cAVR group had infective endocarditis. Despite this, in-hospital mortality was observed in only one out of the eight patients with prosthetic valve endocarditis in the su-AVR group, while in-hospital mortality was not observed in any of the nine endocarditis patients in the cAVR group. These findings suggest that the use of su-AVR for redo cases with prosthesis infective endocarditis is effective in achieving acceptable short- and medium-term survival, as reported in recent literature.

### Echocardiographic outcomes

Evidence on the status of late hemodynamic in sutureless aortic valve replacement is still needed in the literature[20, 21]. Recent studies have suggested no significant hemodynamic differences early period after surgery between sutureless valves and conventional bioprostheses[20, 21]. This observation extends, although with limited evidence, to redo aortic valve surgeries[22].

Our study aimed to address this gap through a meticulous echocardiographic assessment, evaluating sutureless aortic valve prostheses in redo cases. With a median 24-month follow-up, our findings indicate no notable differences between conventional AVR (cAVR) and sutureless AVR (su-AVR) regarding aortic valve median gradient and peak gradient. These measurements remain well within acceptable clinical ranges for both groups.

#### Strengths and limitations

This study yields critical insights into the off-label utilization of a pivotal prosthetic technology within a high-risk patient demographic. Consequently, the sample size is relatively modest compared to most investigations examining outcomes in aortic valve surgery, necessitating prudence in result interpretation. Notably, the patient cohort displays heterogeneity due to diverse comorbid conditions, such as prosthetic valve endocarditis, and variable prior index procedures.

A salient limitation lies in the meticulous selection of the patient group from a singular center, where procedures were executed by a proficient aortic team. This circumstance curtails the generalizability of the findings. Furthermore, the sample population did not attain a size conducive to comprehensive subgroup analysis, rendering caution in the broad application of regression test results. It is, therefore, imperative that forthcoming studies encompass more expansive cohorts to facilitate in-depth analyses, ensuring precise identification of potential disparities in outcomes between the two treatment modalities.

## Conclusion

The findings of this study suggest that Perceval aortic valve replacement can be safely used in patients undergoing redo cardiac surgery, with equivalent outcomes to conventional sutured valves. This is particularly relevant in the context of prosthetic valve endocarditis, a severe and life-threatening form of endocarditis that affects patients with prosthetic heart valves. The compounded effect of both redo surgery and an infected prosthesis can seriously hamper patient survival in the acute setting, making this an area of significant clinical need. There are several potential benefits of using sutureless aortic valves in high-risk patient populations, and the present study suggests that the use of Perceval valves may be an attractive option for patients undergoing redo cardiac surgery. Further research is needed to confirm these findings and to identify the optimal patient selection criteria for the use of sutureless aortic valves in this context.

## Data Availability

The data that supports the findings of this study are available upon request. Due to the sensitive nature of the data and the confidentiality agreements in place, we are unable to publicly share the data directly. However, we are committed to promoting transparency in scientific research, and we encourage interested researchers to contact Corresponding Author to request access to the data.
